# Clinical and Mechanistic Evidence for Comano Thermal Water: A Narrative Review

**DOI:** 10.3390/ijms27093893

**Published:** 2026-04-27

**Authors:** Ermanno Baldo, Damiano Abeni, Giovanni Agostini, Ubaldo Armato, Paolo Bauer, Anna Belloni Fortina, Anna Calza, Elisa Cervadoro, Anna Chiarini, Giorgio Ciprandi, Ilaria Dal Prà, Angela Faga, Stefania Farina, Davide Geat, Mattia Giovannini, Giampiero Girolomoni, Paolo Gisondi, Olivier Jousson, Serena Manara, Eugenio Mira, Giovanni Nicoletti, Calogero Pagliarello, Renato Pedron, Anna Peroni, Vittoria Rizzo, Nicola Segata, Glenda Tettamanti, Mauro Zanoni, Giuseppe Zumiani, Mario Cristofolini

**Affiliations:** 1 G.B. Mattei Institute for Research in Medical Hydrology and Thermal Medicine, 40138 Trento, Italy; 2 Italian Society of Paediatric Allergy and Immunology (SIAIP), 20126 Milano, Italy; 3 IDI—IRCCS Istituto Dermopatico dell’Immacolata, 00167 Rome, Italy; 4 School of Specialisation in Medical Hydrology, University of Pisa, 56126 Pisa, Italy; 5 Section of Maxillofacial Surgery and Dentistry, Department of Surgery, Dentistry, Pediatrics & Gynecology, University of Verona, 37137 Verona, Italy; 6 Independent Dermatologist, 38100 Trento, Italy; 7 Department of Women’s and Children’s Health, University of Padua, 35122 Padua, Italy; 8 Azienda Provinciale per i Servizi Sanitari (APSS), 38123 Trento, Italy; 9 Dermatology and Melanoma Unit (Unità Operativa Complessa—UOC), Ospedale Civile di Livorno, 57124 Livorno, Italy; 10 Department of Medicine and Health Sciences, University of Molise, 86100 Campobasso, Italy; 11 Advanced Technologies for Regenerative Medicine and Inductive Surgery Interdepartmental Research Center T.A.Me.Ri.C.I., University of Pavia, 27100 Pavia, Italy; 12 Comano Thermal Spa Consortium, 38070 Trento, Italy; 13 Dermatology Unit, Azienda Provinciale per i Servizi Sanitari (APSS) Trento, 38123 Trento, Italy; 14 Allergy Unit, Meyer Children’s Hospital IRCCS, 50139 Florence, Italy; 15 Department of Health Sciences, University of Florence, 50139 Florence, Italy; 16 Section of Dermatology, Department of Medicine, University of Verona, 37129 Verona, Italy; 17 Department of Cellular, Computational and Integrative Biology (CIBIO), Centre for Medical Sciences (CISMed), University of Trento, 38122 Trento, Italy; 18 Laboratory of Computational Metagenomics, Department of Cellular, Computational and Integrative Biology (CIBIO), University of Trento, 38122 Trento, Italy; 19 Fondazione I.R.C.C.S. Policlinico San Matteo, University of Pavia, 27100 Pavia, Italy; 20 Department of Clinical, Surgical, Diagnostic and Paediatric Sciences, University of Pavia, 27100 Pavia, Italy; 21 Surgery Unit, ASST (Azienda Socio-Sanitaria Territoriale) di Pavia, 27100 Pavia, Italy; 22 Integrated Unit of Experimental Surgery, Advanced Microsurgery and Regenerative Medicine, University of Pavia, 27100 Pavia, Italy; 23 Division of Dermatology, ASUIT (Azienda Sanitaria Universitaria Integrata del Trentino), 38123 Trento, Italy; 24 Department of Cellular, Computational and Integrative Biology (CIBIO), University of Trento, 38122 Trento, Italy; 25 Dermatology Clinic, University of Verona, 37129 Verona, Italy; 26 Department of Molecular Medicine, University of Pavia, 27100 Pavia, Italy; 27 ASST (Azienda Socio-Sanitaria Territoriale) of Pavia, 27100 Pavia, Italy; 28 Independent Dermatologist, 38122 Trento, Italy

**Keywords:** Comano thermal water, balneotherapy, psoriasis, atopic dermatitis, skin inflammation, immunomodulation, skin barrier, microbiome, photobalneotherapy

## Abstract

Comano thermal water (CTW) is a hypotonic, bicarbonate–calcium–magnesium mineral water traditionally used to manage chronic inflammatory and relapsing skin diseases. This review summarises and discusses the available clinical, experimental, and translational evidence on CTW, with a particular focus on dermatological indications. The physicochemical properties of CTW, along with the presence of a stable, non-pathogenic microbial community, are examined in relation to their potential biological activity. Clinical studies indicate that CTW-based balneotherapy, alone or in combination with narrowband Ultraviolet B (UVB) phototherapy, is associated with improvements in disease severity, symptom burden, and quality of life in patients with psoriasis and atopic dermatitis, and has a favourable safety and tolerability profile. Experimental data further suggest that CTW may exert anti-inflammatory and immunomodulatory effects, modulate keratinocyte function, support skin barrier restoration, and influence the cutaneous microenvironment, including microbiome-related pathways. The review also outlines emerging evidence for CTW in skin regeneration and in upper airway inflammatory conditions treated via inhalation-based approaches. Overall, this review suggests that CTW may serve as a biologically active therapeutic resource, warranting further investigation as a complementary approach within integrative management strategies for inflammatory and barrier-related conditions.

## 1. Introduction

Comano Thermal Water Centre, located in Trentino, Italy, is a historic thermal spa whose use for health purposes can be traced back to Roman times. Archaeological excavations conducted in the 1970s during construction of the modern thermal facility uncovered coins from the Augustan era, water channels, and bathing pools, confirming the site’s long-standing therapeutic use. Its modern development is closely associated with Giovan Battista Mattei, who in the 19th century recognised the waters’ therapeutic potential, acquired the property, and established the first structured thermal baths, with particular emphasis on accessibility. At his death in 1826, Mattei bequeathed the spa to the valley’s poor, establishing a philanthropic legacy that continues today through the allocation of a portion of the spa’s annual profits to charitable activities.

The Comano thermal water is bicarbonate–calcium–magnesium in composition, with a near-neutral pH and a source temperature of approximately 26–28 °C. Its biological and anti-inflammatory properties have been investigated in experimental and clinical studies, and recent microbiological analyses have identified a previously unknown microorganism, *Mesorhizobium comanense*, as part of the stable, non-pathogenic microbial community of the spring. While the precise contribution of this microorganism to clinical effects remains under investigation, its identification has prompted further interest in the water’s potential biological activity beyond its mineral composition. Comano Thermal Water Centre primarily focuses on dermatological conditions, particularly psoriasis and atopic dermatitis.

The use of thermal waters for therapeutic purposes has a long tradition, with balneotherapy practiced worldwide as an adjunctive treatment for chronic skin diseases, musculoskeletal disorders, and respiratory conditions [1,2]. In recent decades, thermal medicine has increasingly been examined through an evidence-based framework, supported by clinical, experimental, and translational research [3]. At Comano, this scientific commitment is reinforced by the dedicated research institute, the Istituto G.B. Mattei per la ricerca in idrologia medica e medicina termale, which investigates treatment efficacy and coordinates clinical and laboratory investigations into treatment outcomes and mechanisms of action. Clinical studies have consistently shown improvements in disease severity, skin barrier function, and quality of life in patients undergoing thermal therapy [4].

This review aims to synthesise and critically discuss the available evidence on the therapeutic use of Comano thermal water, with a primary focus on dermatological disorders such as psoriasis and atopic dermatitis. Additional applications, including upper airway inflammatory conditions and skin regeneration, are also examined, acknowledging differences in the strength and maturity of the available evidence. By integrating data on physicochemical properties, clinical outcomes, and proposed biological mechanisms, this review seeks to clarify the role of Comano thermal water as a complementary, non-pharmacological intervention within contemporary, multidisciplinary medical practice.

## 2. Overview of Comano Thermal Water (CTW)

Comano thermal water (CTW) is a hypotonic, bicarbonate–calcium–magnesium mineral water with unique physicochemical properties contributing to its therapeutic effects [5]. It has a near-neutral pH (7.2–7.55), a low buffer capacity, and low mineralisation (dry residue 190 mg/L, specific electrical conductivity 282–308 µS/cm), making it particularly suitable for dermatological applications (Table 1) [3,5,6]. The water is rich in fluoride and contains relatively high concentrations of bicarbonate, calcium, and magnesium, which play roles in skin barrier function and inflammation modulation [3,7]. The water emerges at the Antica Fonte spring at a temperature of approximately 26–28 °C. Analytical data from two independent time points (2000 and 2012) confirm the compositional stability of CTW over time (Table 1) [3,7].

**Figure 1:**
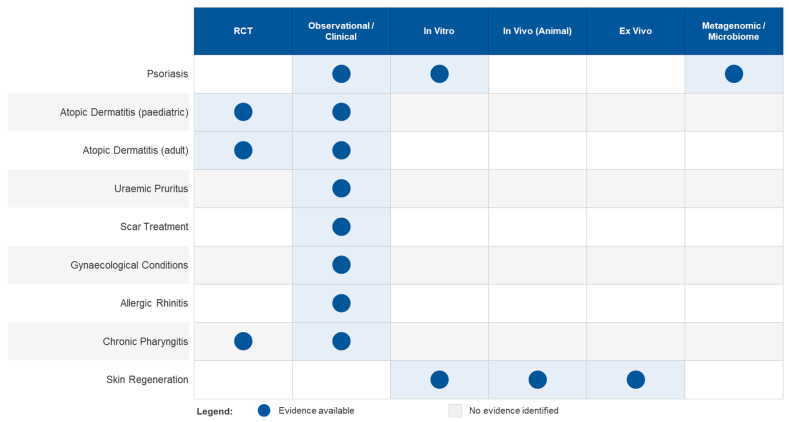
Evidence map for the therapeutic applications of Comano thermal water. Filled circles indicate that experimental or clinical evidence is available for the corresponding combination of indication (rows) and study type (columns); empty cells indicate that no evidence has been identified.

Recent studies have explored the microbiome of Comano thermal water, identifying a stable microbial community dominated by non-pathogenic bacteria [9,10,11]. The core microbiome includes bacterial groups such as *Sphingomonadales*, *Rhizobiales*, and *Caulobacterales*, which are known for their environmental stability and potential immunomodulatory effects [9,12]. These microbial populations may contribute to the water’s therapeutic properties, particularly in promoting skin regeneration and restoring microbial balance in inflammatory skin conditions [11,13] (Figure 1).

The anti-inflammatory properties of Comano thermal water are well-documented, with research demonstrating its ability to downregulate key inflammatory mediators, including interleukin-6 (IL-6), tumour necrosis factor-alpha (TNF-α), and interleukin-8 (IL-8) [14]. Experimental models have also shown that exposure to Comano thermal water promotes keratinocyte proliferation and migration, supporting its role in wound healing and skin regeneration [5].

Comano thermal water is supplied following the principles of minimal treatment to preserve its natural composition [12]. This approach ensures the water remains bacteriologically pure while avoiding interventions that could alter its mineral content, temperature, or microbiome, thereby maintaining its therapeutic properties. Rigorous quality control measures guarantee that the water reaches patients in its natural state, ready for therapeutic use [12].

## 3. Psoriasis

Psoriasis is a chronic, systemic, immune-mediated disease that primarily affects the skin, presenting as red, inflamed, itchy, and scaly plaques, typically on the elbows, knees, scalp, and lower back [15,16]. The condition affects 2–3% of the global population, with onset often occurring in adulthood. Despite advances in treatment, there is no disease-modifying therapy, and long-term management relies on controlling clinical manifestations rather than altering the disease course. Current therapeutic approaches include topical treatments, systemic medications, and phototherapy, which can be effective but often have limitations, such as adverse effects, loss of efficacy over time, or the need for continuous treatment.

Balneotherapy with thermal spring water has emerged as a non-pharmacological option for psoriasis management, offering a well-tolerated and natural approach to signs and symptom relief [15]. At Comano Thermal Water Centre, thermal treatments have been studied for their ability to reduce inflammation, restore skin homeostasis, and improve patients’ quality of life.

### 3.1. Balneotherapy and Photobalneotherapy for Psoriasis

Clinical studies have shown that balneotherapy and photo balneotherapy (balneotherapy combined with narrow-band UVB phototherapy) can significantly improve Psoriasis Area and Severity Index (PASI) scores in patients with chronic plaque psoriasis [1]. A study conducted at the Comano Thermal Water Centre found that one or two weeks of balneotherapy resulted in a statistically significant reduction in PASI scores, with two weeks yielding the greatest improvement. The most pronounced benefits were observed in patients undergoing photo balneotherapy, where PASI scores decreased by nearly 20% after two weeks (*p* < 0.005) [1].

An earlier controlled, randomised, double-blind trial comparing 12 CTW baths with tap water baths in 50 patients with psoriasis reported clinical improvement in 64.4% of CTW-treated patients versus 11.5% in the tap water group (*p* < 0.001), with significant reductions in hyperkeratosis, acanthosis, and papillomatosis on histological examination [3].

The standard balneotherapy course at Comano consists of 12 baths in thermal water, with an additional recommendation to drink approximately 1 litre of thermal water per day on an empty stomach; however, the independent contribution of oral intake to clinical outcomes has not been specifically evaluated in controlled studies. Treatment periods typically last 1–2 weeks [1,15]. Some clinical observations suggest that improvements in disease severity and quality of life may persist beyond the active treatment period, although further studies with defined relapse and remission endpoints are needed to confirm long-term outcomes [4].

### 3.2. Mechanisms of Action in Psoriasis

The beneficial effects of Comano thermal water in psoriasis have been linked to its ability to modulate the skin and gut microbiome, regulate immune responses, and reduce inflammation. Emerging research suggests that balneotherapy may promote significant changes in the skin microbiome of patients with psoriasis. A study using amplicon sequencing analysis revealed that, after a 12-bath balneotherapy course at Comano Thermal Water Centre, the microbiome composition of psoriatic skin shifted to resemble that of unaffected skin, indicating a partial restoration of microbial balance [15]. This observational study included 57 adult patients with different manifestations of psoriasis, including plaque, guttate, and inverse forms. Among the 43 patients with evaluable samples, 67.4% showed a clinical improvement in PASI and/or BSA scores after treatment (*p* <  0.05, Fisher’s exact test). The study also identified low-abundance bacterial biomarkers associated with disease status and treatment efficacy, further supporting the hypothesis that the skin microbiome contributes to psoriasis severity. Additionally, metagenomic sequencing demonstrated that drinking Comano thermal water modifies the gut microbiome, increasing species associated with favourable metabolic health [15]. Notably, 25 gut microbial species were differentially abundant before and after treatment, with several species positively associated with metabolic health showing a significant increase. These findings suggest that balneotherapy has both local and systemic effects, impacting the skin directly and exerting systemic effects through the gut–skin axis.

In addition to modulating the microbiome, Comano thermal water exerts potent anti-inflammatory effects by modulating key inflammatory mediators implicated in psoriasis. In vitro studies by Chiarini et al. have demonstrated that exposure to CTW significantly downregulates vascular endothelial growth factor A (VEGF-A), a key player in the angiogenic and inflammatory cascade that characterises psoriatic plaques [6]. VEGF-A is overexpressed in both lesional and non-lesional skin of psoriasis patients, and elevated circulating levels closely mirror disease activity. Produced primarily by epidermal keratinocytes, VEGF-A promotes endothelial cell proliferation, increased vascular permeability, and leukocyte chemotaxis, which are hallmarks of the aberrant dermal angiogenesis observed in psoriasis [17]. Elevated VEGF expression is also associated with early-onset psoriasis and psoriatic arthritis. In animal models, overexpression of VEGF-A leads to the development of psoriasiform lesions, which resolve upon VEGF inhibition [17]. The ability of Comano thermal water to reduce VEGF-A levels thus aligns with the therapeutic rationale of targeting angiogenesis in the management of psoriasis.

Similarly, Comano water has been shown to suppress interleukin-6 (IL-6) production, another pivotal cytokine in psoriasis pathogenesis [18]. IL-6 contributes to keratinocyte hyperproliferation, promotes Th17 differentiation, and inhibits regulatory T-cell activity. Elevated baseline serum IL-6 has been independently associated with poor treatment response and early discontinuation of systemic therapies in real-world psoriasis cohorts [19]. Although IL-6 may not be the principal effector in cutaneous inflammation, its systemic effects, primarily on immune regulation and metabolic dysfunction, underscore its role as a clinically relevant biomarker of disease burden and treatment sustainability. The 2006 in vitro study by Chiarini et al. conducted on keratinocytes isolated from six adult patients with psoriasis demonstrated that exposure to Comano thermal water significantly reduced both intracellular IL-6 levels (−73% at day 3, *p* < 0.001) and its secretion (−88% to −96% by day 15, *p* < 0.001). Moreover, IL-6 secretion was normalised with as little as 25% Comano water in the culture medium, indicating a strong dose-dependent effect [18]. Balneotherapy with Comano thermal water may, therefore, offer dual local and systemic benefits by modulating both VEGF-A and IL-6, mitigating inflammatory angiogenesis, and supporting long-term immune homeostasis. Further evidence suggests that Comano thermal water suppresses the expression of tumour necrosis factor-alpha (TNF-α) and interleukin-8 (IL-8), two key pro-inflammatory cytokines that drive immune activation and neutrophil recruitment in psoriatic lesions [20]. Moreover, Cytokeratin-16 (CK-16), a marker of abnormal keratinocyte differentiation and hyperproliferation in psoriasis, is downregulated following thermal water exposure, suggesting a direct effect on epidermal cell function [18].

**Figure 2:**
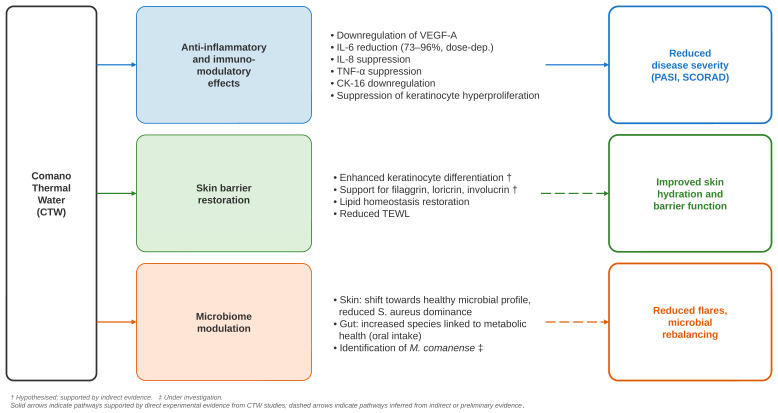
Proposed mechanisms of action of Comano thermal water (CTW) in inflammatory skin diseases. Three converging pathways are illustrated: anti-inflammatory and immunomodulatory effects (reducing disease severity, as measured by PASI and SCORAD), skin barrier restoration (improving hydration and barrier function), and microbiome modulation (promoting microbial rebalancing and fewer flares). Cytokine and growth-factor effects supporting the anti-inflammatory pathway include downregulation of VEGF-A [6], suppression of IL-6 (73–96%, dose-dependent) and CK-16 [18], and suppression of IL-8 and TNF-α [20]. The barrier-restoration pathway is supported by reduced trans-epidermal water loss and histological evidence of enhanced dermal architecture [5]. The microbiome-modulation pathway is supported by metagenomic evidence of reduced *S. aureus* dominance and a healthier cutaneous profile [9,13], gut microbial rebalancing following oral intake [13], and identification of Mesorhizobium comanense in the spring water [10]. † Hypothesised; supported by indirect evidence. ‡ Under investigation. Solid arrows indicate pathways supported by direct experimental evidence from CTW studies; dashed arrows indicate pathways inferred from indirect or preliminary evidence.

Taken together, these findings suggest that CTW-based balneotherapy may act through multiple pathways in psoriasis, including microbiome modulation, suppression of pro-inflammatory cytokines, and normalisation of keratinocyte function. The current evidence, derived from observational clinical studies and in vitro experiments, supports further investigation of CTW as a complementary, non-pharmacological approach within psoriasis treatment strategies (Figure 2). Further studies are needed to better understand the microbiome’s role and other underlying mechanisms in the therapeutic effects of balneotherapy.

### 3.3. Impact on Quality of Life and Patient-Reported Outcomes

Balneotherapy at the Comano Thermal Water Centre has been shown to improve both self-reported psoriasis severity, as measured by the Self-Administered Psoriasis Area and Severity Index (SAPASI), and quality of life, as assessed by the Skindex-17 questionnaire [22]. The treatment significantly reduced disease severity, with improvements observed in both physical and psychological aspects of psoriasis [4]. Patients with psoriasis often self-refer to Comano Thermal Water Centre, demonstrating a positive perception of this treatment modality [23]. In a study comparing psoriasis patients attending Comano Thermal Water Centre with those receiving hospital-based care, spa patients were more likely to prefer balneotherapy [23]. This preference, as reported in the study, was partly driven by concerns about the side effects of pharmacological therapies. Additionally, the Italian health system partially covers the cost of balneotherapy, making it an accessible option for many patients.

## 4. Atopic Dermatitis

Atopic dermatitis (AD) is a common inflammatory skin condition that primarily affects children. It is characterised by chronic, relapsing episodes of erythema, pruritus, and skin barrier dysfunction, often leading to significant discomfort and reduced quality of life [24]. Management typically involves the use of emollients to restore the skin barrier, topical corticosteroids (TCS) and calcineurin inhibitors (TCI) to control inflammation, and, in severe cases, systemic treatments such as immunosuppressants or biologic therapies. However, long-term disease control remains challenging, as many patients experience recurrent flares, and patients’ concerns about side effects, particularly with corticosteroids, can lead to suboptimal adherence [25].

### 4.1. Balneotherapy for AD in Children

Balneotherapy has long been used as a supportive treatment for inflammatory skin diseases, including atopic dermatitis (AD), offering a natural and well-tolerated approach to symptom management [24]. At Comano Thermal Water Centre, balneotherapy involves full-body immersion in individual bathtubs for 2–20 min, once or twice daily, over the course of 12–24 baths, typically spanning one to two weeks. During this period, patients are advised to apply emollients once daily after bathing, while avoiding other topical or systemic treatments, to allow for a more precise assessment of the water’s therapeutic effects [16]. A randomised clinical trial involving 104 children with mild-to-moderate AD demonstrated significant clinical improvements following balneotherapy at Comano. The study found that patients treated with thermal water experienced a marked reduction in AD severity scores (SCORAD) and improvements in investigator global assessment (IGA), pruritus intensity, and quality-of-life indices [16]. Notably, while TCS provided a more immediate reduction in disease severity, children who underwent balneotherapy experienced sustained benefits, with fewer relapses and longer symptom-free periods compared with those treated with corticosteroids alone. Further supporting these findings, an extensive observational study by Geat et al. analysed 867 children with atopic dermatitis treated with balneotherapy at Comano Thermal Water Centre over a single season [25]. The cohort included children aged ≤16 years (mean age 5.9 years), with 41.2% presenting mild AD, 43.6% moderate, and 15.2% severe disease based on SCORAD. The study found that early-onset AD (particularly before 6 months of age), severe disease, and coexisting food allergies were significantly associated with more severe clinical presentation and, conversely, with greater clinical improvement following treatment. Children with severe AD were also more likely to have followed elimination diets, although only 27.2% of these had a documented food allergy, indicating a possible overuse of dietary restriction. Treatment patterns revealed that while emollients (55.1%) and topical corticosteroids (45.7%) were the most common therapies used before admission, use of systemic treatments was limited, and undertreatment was notable even in severe cases—9.8% of children with severe AD had not used TCS, TCI, or systemic therapy in the month before attending the spa. The authors observed that balneotherapy at Comano may be particularly beneficial in this under-treated population, offering a safe, non-pharmacological option for symptom relief. Although the study did not include post-treatment SCORAD follow-up, its comprehensive profiling of disease characteristics and management patterns provides strong support for the use of thermal water therapy in paediatric AD, especially in those with early-onset or comorbid allergic conditions.

### 4.2. Balneotherapy for AD in Adults

Beyond Comano, similar beneficial effects of thermal treatments have been reported at other European centres, such as Avène in France, and the Dead Sea in Israel [26,27]. A large observational study of over 5000 adult patients undergoing a three-week hydrotherapy programme at Avène showed significant reductions in SCORAD scores among individuals with atopic dermatitis (AD), supporting the role of balneotherapy in managing this condition [26]. Likewise, climatotherapy at the Dead Sea has been shown to improve AD signs and symptoms and can be used as an adjunctive treatment option [27]. Further supporting these findings, a controlled, double-blind, randomised trial conducted at Comano (April–October 1998) enrolled 50 patients with eczematous dermatitis (atopic and contact forms) and compared 12 CTW baths with tap water baths under identical conditions of temperature (37 °C) and duration (20 min) [8]. The mean clinical evaluation index showed a 69.1% improvement in the CTW group versus 29.7% in the tap water group (*p* = 0.002), with 52% of patients achieving excellent results. Cutaneous hydration improved significantly only in the CTW group, while no adverse effects were reported. These findings were subsequently included in a 2024 systematic review of balneotherapy for dermatological diseases [8,28]. In addition to whole-body bathing, thermal spring components have also been incorporated into topical formulations. An observational study in 1399 adults with atopic dermatitis or xerotic skin disorders assessed an “emollient plus” containing non-living lysates of *Vitreoscilla filiformis* bacteria from La Roche-Posay Thermal Spring Water. After two months of use, more than 90% of patients reported significant improvement in disease intensity, pruritus, dryness, and quality of life (*p* <  0.001). These findings support the broader application of thermal spring-derived therapies for adults with AD, whether through immersion or topical delivery [29].

### 4.3. Guidelines and Recommendations

National and international guidelines have further validated the clinical relevance of balneotherapy in the treatment of atopic dermatitis (AD). The 2016 Consensus Conference, led by Galli et al., highlighted the role of non-pharmacological treatments, including thermal therapies, in improving disease control in children with AD. It strongly emphasised therapeutic education and structured interventions as central to long-term management. This aligns closely with the model adopted at Comano, where initiatives such as the School of Atopy combine clinical care with caregiver training and psychological support [30]. The 2018 European guidelines for the treatment of atopic eczema, developed by the European Dermatology Forum (EDF), the European Academy of Dermatology and Venereology (EADV), and collaborating groups, include thermal water balneotherapy as a complementary treatment option that may be integrated into a broader management plan. The guidelines support daily bathing followed by emollient application as a core part of basic care and acknowledge that selected non-pharmacological approaches, such as balneotherapy, can be considered based on patient preference and clinical context [31]. The 2020 ETFAD/EADV position paper, issued by the European Task Force on Atopic Dermatitis and endorsed by EADV, builds on this integrated care framework, emphasising the value of non-pharmacological strategies alongside pharmacological treatments. These include patient education, psychological support, and lifestyle-oriented interventions. Although balneotherapy is not explicitly detailed, the document encourages structured, multidisciplinary approaches that align with spa-based programmes such as those offered at Comano, particularly when incorporating education, adherence support, and validated outcome monitoring [32]. More recently, the 2022 Italian guidelines by Galli et al., developed collaboratively by the Italian Society of Paediatric Allergy and Immunology (SIAIP), the Italian Society of Paediatric Dermatology (SIDerP), and the Italian Society of Paediatrics (SIP), endorsed the integration of non-pharmacological interventions, including balneotherapy, within a multidisciplinary care pathway for moderate to severe paediatric AD [33]. These guidelines emphasise the need to tailor treatment to clinical phenotype and to consider spa therapy as an adjuvant, particularly when aiming to reduce pharmacologic burden or manage flares.

### 4.4. Patient Education and Long-Term Management

The therapeutic impact of balneotherapy at Comano Thermal Water Centre extends well beyond short-term clinical improvements. Central to its long-term strategy is the School of Atopy, a structured educational programme embedded within the spa’s care model. Operational for over 15 years, this initiative is grounded in the principles of therapeutic education, as defined by the World Health Organization, which aims to empower patients and families to acquire the skills necessary to manage chronic conditions effectively.

At Comano, the School of Atopy delivers weekly multidisciplinary sessions throughout the thermal season, targeting both the child and their caregivers. These sessions are led by a team comprising dermatologists, allergists, psychologists, and educators who employ interactive methodologies, such as cooperative learning, problem-solving, role-playing, and hands-on learning. Parents are actively engaged in understanding disease mechanisms, treatment principles, and daily management techniques, including the application of emollients, the use of wet wraps, and adherence strategies. Notably, the sessions also include facilitated psychological consultations aimed at addressing the emotional burden of disease, including stress, sleep disruption, and feelings of inadequacy often experienced by parents. Emerging needs, such as coping strategies and peer support, are addressed, helping caregivers shift from a passive to an empowered role [34]. National allergy and dermatology societies have recognised this educational model, and it serves as a blueprint for multidisciplinary, family-centred care in chronic paediatric diseases. It encourages knowledge sharing, emotional validation, and shared problem-solving, ultimately fostering greater control and resilience among families affected by AD. To support home-based disease monitoring and continuity of care, Comano Thermal Water Centre has also introduced the Comano Score—a caregiver-reported global severity measure developed and validated specifically for paediatric atopic eczema. Using a simple 0–10 numerical rating scale, this tool enables parents to rapidly and intuitively rate their child’s disease severity. In a cross-sectional study involving 867 children undergoing balneotherapy, the Comano Score demonstrated a strong correlation with the physician-assessed SCORAD (r = 0.74, *p* < 0.0001), highlighting its potential as a reliable, user-friendly outcome measure in both clinical and home settings [35]. The School of Atopy and the Comano Score exemplify Comano’s commitment to a comprehensive, patient-centred model of care that combines clinical treatment, therapeutic education, and caregiver empowerment to improve both short- and long-term outcomes in paediatric AD.

### 4.5. Mechanisms of Action in AD

The therapeutic effects of Comano thermal water in atopic dermatitis are supported by its immunomodulatory, anti-inflammatory, and microbiome-modulating properties. The water’s impact on cytokine production and the thermal spring’s unique microbial composition are two key mechanisms contributing to its beneficial effects.

Comano thermal water has been shown to interfere with key inflammatory mediators involved in AD, particularly interleukin-6 (IL-6), interleukin-8 (IL-8), and tumour necrosis factor-alpha (TNF-α). These cytokines drive immune activation and inflammation in AD, contributing to keratinocyte hyperproliferation, pruritus, and skin barrier dysfunction [18,20]. Studies on psoriatic keratinocytes indicate that exposure to Comano thermal water reduces the production and secretion of these cytokines, suggesting the downregulation of inflammatory signalling pathways that may extend to AD pathology [6,18]. Additionally, TNF-α, a cytokine heavily implicated in AD pathogenesis, is significantly suppressed by Comano thermal water, further reinforcing its potential to mitigate chronic inflammation and reduce disease severity [20]. These findings suggest that Comano thermal water helps restore immune balance in the skin, reducing inflammation and improving signs and symptoms in patients with AD.

Dysbiosis, particularly the overgrowth of *Staphylococcus aureus,* is critical in exacerbating AD clinical manifestations and compromising skin barrier function [36]. Comano thermal water contains a unique microbial ecosystem, with dominant bacterial taxa including *Sphingomonadales*, *Rhizobiales*, and *Caulobacterales* orders, as well *as Bradyrhizobiaceae* and *Moraxellaceae* families [12]. A key recent discovery is the identification of *Mesorhizobium comanense*, a bacterial species isolated from Comano thermal water with potential anti-inflammatory properties [10]. Although further research is needed to fully elucidate its role, preliminary findings suggest that *Mesorhizobium comanense* may help regulate immune responses and promote microbial balance, making it a promising contributor to the therapeutic effects of thermal water. The skin microbiome in AD patients is often characterised by reduced microbial diversity, which weakens the skin’s natural defence against pathogens and increases susceptibility to flares and secondary infections [37]. Balneotherapy with Comano thermal water may help promote a more balanced skin microbiome, limiting *S. aureus* colonization and supporting the growth of beneficial bacterial species.

AD is strongly associated with epidermal barrier dysfunction, driven by abnormalities in lipid metabolism and structural proteins [38]. The stratum corneum lipid matrix, which consists primarily of ceramides, free fatty acids, and cholesterol, is essential for maintaining skin hydration and protection against external allergens and irritants. In AD, ceramide levels are significantly reduced, leading to increased transepidermal water loss (TEWL) and skin barrier fragility [38]. Recent research suggests that cytokine dysregulation, particularly IL-4 and IL-13, contributes to ceramide depletion by inhibiting key lipid metabolism enzymes [37]. Through its anti-inflammatory properties, Comano thermal water may help counteract these effects by restoring lipid homeostasis and supporting the reorganisation of the skin barrier. By attenuating the cytokine-driven disruption of keratinocyte differentiation, balneotherapy could theoretically support the production of structural proteins such as filaggrin (FLG), loricrin, and involucrin, which are essential for epidermal integrity, although direct evidence from CTW-specific studies is currently lacking [38]. Given that FLG deficiency is a significant risk factor for AD, these findings suggest that thermal treatments may help mitigate barrier defects and improve overall skin resilience.

### 4.6. Future Directions

While existing evidence strongly supports the anti-inflammatory, microbiome-modulating, and barrier-restoring effects of Comano thermal water, further research is needed to elucidate the molecular mechanisms driving these benefits entirely. Future studies should focus on investigating the long-term impact of thermal treatments on microbial diversity and immune function in AD patients; elucidating the roles of *Mesorhizobium comanense* and other microbial species in immune regulation and skin barrier repair; and exploring the molecular interactions between Comano thermal water and lipid metabolism pathways in the epidermis.

## 5. Other Dermatological Conditions

### 5.1. Scar Treatment

Balneotherapy with Comano thermal water has been explored as a therapeutic option for hypertrophic scars and keloids, offering a natural, non-invasive approach to improving scar texture and elasticity. Hypertrophic scars and keloids result from an excessive fibrotic response during wound healing, leading to excessive collagen deposition, inflammation, and impaired skin remodelling. Traditional treatments, such as intralesional corticosteroids, laser therapy, and surgical excision, can be effective but often pose risks of recurrence and side effects [21].

Thermal rehabilitation at Comano has shown promising clinical benefits in patients with post-surgical, post-burn, and post-traumatic scars. Regular immersion in Comano thermal water has been observed to reduce scar thickness, improve elasticity, and enhance the overall appearance of scars. These effects are attributed to the water’s lenitive, anti-inflammatory, and neoangiogenic properties, which promote tissue remodelling, microcirculation, and fibroblast activity. The bicarbonate–calcium–magnesium composition of the water plays a key role in modulating inflammatory pathways and supporting epidermal regeneration, contributing to a more physiological scar maturation process [39].

### 5.2. Uremic Pruritus

Uremic pruritus is a common and often debilitating symptom in patients undergoing chronic dialysis, significantly impairing quality of life. The underlying mechanisms are complex and not yet fully understood, but they are believed to involve immune dysregulation, inflammation, and alterations in the epidermal barrier. Conventional treatments, including topical emollients, antihistamines, and systemic immunosuppressants, often provide limited relief, necessitating complementary therapeutic approaches [40]. Balneotherapy with Comano thermal water has been investigated as a potential treatment for uremic pruritus. A clinical observational study conducted at the Comano Thermal Water Centre evaluated the effects of balneotherapy in 18 dialysis patients with chronic pruritus and xerosis. Patients underwent a 12-day treatment course consisting of 20-min thermal baths daily and ingested approximately half a litre of Comano thermal water each morning. In this small, uncontrolled observational study, a marked reduction in pruritus severity was reported, with 77.8% of patients experiencing symptom relief and 22.2% achieving complete remission. Skin hydration also improved, reducing xerosis, scratch-induced lesions, and lichenification. Notably, the benefits of balneotherapy persisted for up to two months post-treatment, although the absence of a control group and the small sample size (n = 18) limit the strength of these conclusions [40]. The mechanisms underlying these improvements are not yet fully elucidated, but Comano thermal water’s bicarbonate–calcium–magnesium composition is thought to exert anti-inflammatory, immunomodulatory, and skin-barrier-restoring effects. Balneotherapy may help mitigate the chronic inflammation and skin dysfunction associated with uremic pruritus by reducing pro-inflammatory cytokine production and promoting epidermal hydration. Given its excellent safety profile and lack of systemic side effects, thermal therapy at the Comano Thermal Water Centre represents a promising complementary treatment for dialysis patients with refractory pruritus. Further controlled studies are warranted to confirm these findings and explore potential synergies with other therapeutic modalities, such as UV phototherapy.

### 5.3. Skin Regeneration

Thermal mineral waters have been shown to aid in healing skin irritations and enhance skin restoration following cosmetic procedures that cause epidermal damage [41,42,43]. Their efficacy is likely linked to a combination of hydrating, anti-inflammatory, and barrier-repairing properties, which create a favourable environment for keratinocyte proliferation and dermal remodelling. Comano thermal water has also demonstrated significant potential in promoting skin regeneration, as evidenced by experimental studies. Research by Faga et al. employed an animal wound model using New Zealand White rabbits to assess the effects of Comano thermal water on split-thickness skin-graft donor sites [5]. The study compared the healing response between wounds treated with Comano thermal water, a saline solution, and a standard petrolatum dressing. Histological and ultrastructural analyses revealed that wounds treated with Comano thermal water exhibited faster re-epithelialization, a reduced inflammatory infiltrate, and a more organised dermal architecture than the control groups. By Day 4, the wounds treated with Comano thermal water were fully epithelialised, exhibiting a multilayered epidermis and minimal inflammatory cells, whereas the control wounds lagged in healing [5,42,43]. Further research confirmed these properties in both in vitro human fibroblast cultures and an ex vivo human full-skin experimental model [11,44]. These findings suggest that Comano thermal water accelerates the transition from the inflammatory phase to the proliferative phase of wound healing, supporting earlier tissue remodelling and regeneration. The combined in vivo evidence supports the role of Comano thermal water in enhancing skin regeneration, making it a valuable adjunct in dermatological therapies for conditions characterised by chronic inflammation and impaired wound healing.

## 6. Gynaecological Conditions

Vaginal irrigations with Comano thermal water have been shown to provide therapeutic benefits in patients suffering from various gynaecological conditions, particularly those associated with inflammation and chronic infections. A preliminary prospective study conducted by De Micheli et al. evaluated the effects of 12 consecutive days of vaginal irrigation therapy with Comano thermal water in 43 women affected by chronic inflammatory gynaecological conditions [45]. The study demonstrated significant relief of clinical manifestations, particularly burning, itching, pain, vaginal dryness, dyspareunia, leukorrhea, cystitis, and ulceration. These improvements were observed immediately after treatment and persisted for 30–60 days. The positive effects of Comano thermal water are likely due to its unique mineral composition, which includes bicarbonate, calcium, and magnesium, all of which are known for their anti-inflammatory, decongestant, and tissue-repairing properties. The near-neutral pH of CTW may also support the maintenance of vaginal microbiota balance, although this hypothesis has not been directly tested in microbiological studies. The study also highlighted that the treatment was well tolerated, with high patient compliance and no significant side effects reported [45]. These preliminary findings suggest that vaginal irrigation with CTW may offer symptomatic relief in chronic vaginal conditions, including recurrent vaginitis and vulvovaginal atrophy. However, this was a single-arm study without a control group (n = 43), and the findings require confirmation in larger, controlled trials before firm conclusions can be drawn about the role of CTW in gynaecological care.

## 7. Upper Airway Disorders

Thermal water therapy has long been employed in respiratory medicine, particularly through inhalation-based treatments, with evidence supporting its role in alleviating inflammatory conditions of the upper airways [46,47]. These approaches have traditionally been used in both adults and children, especially in individuals with allergic or hyperreactive airway phenotypes. A recent narrative review by Ciprandi and colleagues further strengthened this evidence, analysing clinical data from six Italian studies involving both adults and children with allergic rhinitis or recurrent respiratory infections. The review reported significant improvements in mucociliary clearance, symptom severity, and infection frequency across treatment groups, suggesting that thermal water inhalations may reduce disease burden and reliance on pharmacological treatments [48]. Collectively, these findings suggest a potential reduction in disease burden and in the need for pharmacological treatments.

In this context, Comano thermal water has been investigated not only for cutaneous indications but also for upper airway disorders treated via inhalation therapy. Historical clinical experience and observational studies describe its use in inflammatory conditions of the nasal and pharyngeal mucosa, particularly in children and in patients with allergic or hyperergic predisposition [49]. The reported benefits include improved mucosal trophism, reduced inflammatory symptoms, and enhanced local defence mechanisms of the respiratory epithelium. These effects have been attributed to the oligomineral composition of Comano thermal water, characterised by bicarbonate, calcium, and magnesium ions, and to its near-neutral pH, which may contribute to mucosal homeostasis. Several studies have specifically examined the effects of Comano thermal water inhalations in patients with allergic rhinitis. A preliminary clinical investigation by Ciprandi and colleagues evaluated adult patients undergoing a cycle of nasal inhalations and observed a significant reduction in nasal obstruction, rhinorrhoea, and overall symptom scores, with benefits persisting beyond the treatment period [50]. A decreased reliance on symptomatic pharmacological therapy was also reported, supporting the role of Comano thermal water inhalations as a complementary, non-pharmacological option.

The mechanisms underlying these effects are likely multifactorial. Thermal water inhalations may improve local blood flow, enhance mucociliary clearance, and support epithelial barrier integrity, thereby reducing allergen permeability across the mucosa. In addition, the mineral content of the water may modulate the local inflammatory response, thereby reducing hypersensitivity and chronic inflammation. Beyond allergic rhinitis, Comano thermal water inhalations have been investigated for the treatment of chronic pharyngitis. A double-blind, controlled trial in 26 patients with chronic pharyngitis compared daily 10-minute nebulised inhalations of Comano thermal water with isotonic saline (NaCl 0.9%) over 15 days [51]. Pharyngeal blood flow and secretory IgA improved significantly only in the CTW group (*p* < 0.05), and subjective improvement was faster and more complete than with saline, supporting the role of Comano thermal water as an adjunctive treatment for chronic upper respiratory tract inflammatory conditions. Although much of this evidence derives from historical and observational studies, it supports the inclusion of inhalation therapies among the broader therapeutic applications of Comano thermal water in inflammatory conditions, alongside its more extensively studied dermatological indications. It should be noted that the strength of the upper airway evidence is limited compared with the dermatological evidence base: the available studies are predominantly uncontrolled, use heterogeneous outcome measures, and lack long-term follow-up. Prospective, controlled trials are needed to establish the efficacy of CTW inhalation therapy in these conditions.

Table 2 and Table 3 provide a consolidated summary of the clinical and experimental evidence reviewed.

## 8. Conclusions

The evidence reviewed in this article supports the therapeutic role of Comano thermal water as a complementary intervention in selected inflammatory conditions, particularly in dermatology. Clinical studies consistently report improvements in disease severity, skin barrier function, and patient-reported outcomes in psoriasis and atopic dermatitis following balneotherapy or photobalneotherapy. These effects are supported by experimental findings indicating anti-inflammatory, immunomodulatory, and microbiome-related mechanisms, although the relative contributions of each pathway require further clarification.

Beyond dermatological indications, available clinical and observational data suggest potential benefits of Comano thermal water for upper airway inflammatory disorders, including allergic rhinitis and chronic pharyngitis, when administered via inhalation. Reported improvements in mucosal function and symptom burden suggest that the therapeutic properties of the water may extend beyond cutaneous applications, although this evidence is more limited and largely derives from non-randomised or historical studies.

Importantly, the body of research reviewed indicates that thermal treatments should be evaluated as structured medical interventions rather than generic “natural” remedies, with defined protocols, outcome measures, and safety profiles. The studies conducted at Comano Thermal Water Centre contribute to this evidence base, demonstrating good tolerability and a favourable safety profile across different patient populations.

Ongoing research conducted by the G.B. Mattei Research Institute continues to investigate the biological mechanisms underlying the observed clinical effects, including immune modulation, barrier restoration, and interactions with microbial communities. Future well-designed controlled studies will be essential for further defining indications, optimising treatment protocols, and clarifying long-term benefits, thereby strengthening the positioning of Comano thermal water within integrative, evidence-based management strategies for chronic inflammatory conditions.

## Figures and Tables

**Table 1:** Physico-chemical composition of Comano thermal water (Antica Fonte spring, Stenico, Trentino, Italy).

Parameter	Value (2000) ^a^	Value (2012) ^b^	Unit
Temperature at source	27.7	26.2	°C
pH	7.2	7.55	—
Specific electrical conductivity (at 18 °C/20 °C)	282	308	μS/cm
Dry residue at 180 °C	190	190	mg/L
Bicarbonate (HCO_3_^−^)	196.56 ^c^	206	mg/L
Calcium (Ca^2+^)	48.9	52.2	mg/L
Magnesium (Mg^2+^)	12.16	14	mg/L
Sodium (Na^+^)	2	1.71	mg/L
Potassium (K^+^)	0.5	0.63	mg/L
Chloride (Cl^−^)	0.8	0.7	mg/L
Sulphate (SO_4_^2−^)	6.9	11.7	mg/L
Silica (SiO_2_)	4.9	6.1	mg/L
Fluoride (F^−^)	0.43	0.4	mg/L
Nitrate (NO_3_^−^)	0.30 ^d^	0.6	mg/L
Strontium (Sr)	0.23	0.21	mg/L
Iron (total/dissolved)	0.01	<0.010	mg/L
Lithium (Li)	0.0006	<0.010	mg/L
Manganese (Mn)	0.0016	<0.02	mg/L
Free carbon dioxide	—	9	mg/L
Oxidisability	—	<0.5	mg/L

^a^ Zumiani G, Zanoni M, Agostini G. G Ital Dermatol Venereol. 2000;135:253–258 (Table I) [8]. ^b^ Provincia Autonoma di Bolzano, Laboratorio analisi acqua 29.5. Rapporto di prova 12LA10521, 19 September 2012 [7] (ACCREDIA-accredited laboratory, LAB N°0434). Sample: Antica Fonte, Terme di Comano, Stenico (TN). Sampling date: 9 August 2012. ^c^ Reported as “ione carbonico” in the original. ^d^ Reported as “ione nitrico” in the original. Classification: hypotonic, hypothermal, bicarbonate–calcium–magnesium mineral water, rich in fluoride.

**Table 2:** Clinical studies on Comano thermal water.

Indication	Study (Design; N)	Comparator/Control	Protocol	Main Outcomes	Limitations
Psoriasis	Peroni et al., 2008 [1]; Observational; N = 300	None (pre–post)	12 baths, 1–2 wk ± NB-UVB	PASI ↓~20% with PBT (*p* < 0.005); confirmed by SAPASI/Skindex-29; lost at 4 mo	No control group; short follow-up
Psoriasis	Zumiani et al., 2000 [3]; Double-blind RCT; N = 50	Tap water	12 baths	Clinical improvement 64.4% (CTW) vs. 11.5% (tap water), *p* < 0.001; significant histological reductions in hyperkeratosis, acanthosis, and papillomatosis	Single-centre; local journal without DOI
Psoriasis (QoL)	Pagliarello et al., 2012 [22]; Observational; N = not reported	Pre–post	BT or balneophototherapy	SAPASI ↓ 27%; Skindex-17 improved; 51% with SAPASI gain had worsened QoL	No DOI; limited detail
Psoriasis (microbiome)	Manara et al., 2023 [15]; Observational; N = 57 (43 eval.)	Pre–post	12 baths	67.4% clinical improvement; skin microbiome shifted to healthy profile; 25 gut species differentially abundant	No control; small sample
Paediatric AD	Farina et al., 2011 [16]; RCT; N = 104 children	TCS	12 baths, 2–20 min, 1–2× daily	SCORAD/IGA: significant improvement; fewer relapses vs TCS; QoL improved	Single-centre
Paediatric AD (profiling)	Geat et al., 2021 [25]; Observational; N = 867 children	None	BT (single season)	Early onset, severe AD, food allergy associated with greater severity; undertreatment noted	No post-treatment SCORAD follow-up
Adult eczematous dermatitis	Zumiani et al., 2000 [8]; Double-blind RCT; N = 48 (50 enrolled, 2 lost to follow-up)	Tap water	12 baths, 20 min, 37 °C, over 20 days; topical emollient permitted	69.1% improvement (CTW) vs 29.7% (tap water); *p* = 0.002; 52% excellent, 36% good; hydration significantly improved in CTW only; no adverse effects	Single-centre; non-validated scoring system; local journal without DOI
Uraemic pruritus	Cattoni et al., 2015 [40]; Observational; N = 18	Pre–post	12 d, 20-min baths + oral intake	77.8% relief; 22.2% complete remission; benefits persisted 2 months	Very small N; no control; local journal
Gynaecological conditions	De Micheli et al., 2016 [45]; Prospective observational; N = 43 women	Pre–post	12 d vaginal irrigation	Significant symptom relief (burning, itching, pain, dryness); persistent 30–60 d	Preliminary; small N; no control
Allergic rhinitis	Ciprandi et al., 2016 [50]; Observational; N = 30	Pre–post	Nasal inhalation, 15 d	TSS significantly decreased; VAS nasal patency improved; benefits at 2-wk follow-up	No control group; preliminary
Chronic pharyngitis	De Paoli et al., 1998 [51]; Double-blind RCT; N = 26	Saline solution (NaCl 0.9%)	Daily inhalation, 1 L via nebuliser, 10 min, 37–38 °C, 15 days	Pharyngeal blood flow and IgAS improved significantly in CTW only (*p* < 0.05); cough resolved in 6/8 CTW vs 5/6 saline; subjective improvement faster and more complete in CTW group	Small sample; single-centre; no DOI; published in Italian only

**Symbols:** ↓ = decrease; **Abbreviations:** AD, atopic dermatitis; BT, balneotherapy; BSA, body surface area; CTW, Comano thermal water; IGA, Investigator Global Assessment; IgAS, secretory immunoglobulin A; NB-UVB, narrowband UVB; PASI, Psoriasis Area Severity Index; PBT, photobalneotherapy; QoL, quality of life; RCT, randomised controlled trial; SAPASI, Self-Administered PASI; SCORAD, SCORing Atopic Dermatitis; SF-36, Short Form-36; TCS, topical corticosteroids; TSS, total symptom score; VAS, visual analogue scale.

**Table 3:** Experimental and molecular evidence for Comano thermal water.

Model/System	Cell/Tissue Type	Exposure	Mediators/Endpoints	Direction of Effect	Reference	Limitations
*In vitro*	Human psoriatic keratinocytes	CTW (various conc.)	VEGF-A protein isoforms	Downregulation of VEGF-A expression and secretion	Chiarini et al., 2006a [6]	Single cell type; no in vivo confirmation
*In vitro*	Human psoriatic keratinocytes (n = 6 donors)	CTW (25–100% in culture medium)	IL-6 (intracellular and secreted); CK-16	IL-6 ↓ 73% (day 3), 88–96% (day 15); CK-16 downregulated; dose-dependent	Chiarini et al., 2006b [18]	Small donor sample; in vitro only
*In vitro*	Human psoriatic keratinocytes	CTW	TNF-α expression; IL-8 production and secretion	Suppression of TNF-α and IL-8	Dal Prà et al., 2007 [20]	*In vitro* only
*In vivo* (animal)	NZW rabbits; split-thickness skin graft donor sites	CTW vs saline vs petrolatum dressing	Re-epithelialisation; inflammatory infiltrate; dermal architecture	Faster re-epithelialisation; reduced inflammation; organised dermis by Day 4	Faga et al., 2012 [5]	Animal model; small sample
*In vitro*	Human skin fibroblasts	Bacterial lysates from CTW spring water	Cell proliferation; regenerative markers	Promotion of fibroblast proliferation and regenerative activity	Nicoletti et al., 2016 [11]	Preliminary; lysates not whole water
*Ex vivo*	Human full-skin model	CTW-derived bacterial lysates	Tissue regeneration; structural integrity	Enhanced skin regeneration	Nicoletti et al., 2023 [13]	*Ex vivo* model; lysates not whole water
Metagenomics	CTW spring water	N/A	Microbial community composition	Stable non-pathogenic community: *Sphingomonadales*, *Rhizobiales*, *Caulobacterales*; identification of *M. comanense*	Pedron et al., 2019 [12]; Pedron et al., 2021 [10]	Descriptive; no direct link to clinical outcomes

**Symbols:** ↓ = decrease; **Abbreviations:** CK-16, cytokeratin-16; CTW, Comano thermal water; IL, interleukin; NZW, New Zealand White; TNF-α, tumour necrosis factor-alpha; VEGF-A, vascular endothelial growth factor-A.

## Data Availability

No new data were created or analysed in this study. Data sharing is not applicable to this article.
